# Supplementary material for: Using high-definition transcranial direct current stimulation to investigate the role of the dorsolateral prefrontal cortex in explicit sequence learning
Source: PLoS One. 2021 Mar 18;16(3):e0246849. doi: 10.1371/journal.pone.0246849 (PMC7971701; doi:10.1371/journal.pone.0246849)
Supplement: Attachment — Submitted filename: Response to Reviewers.docx [file pone.0246849.s002.docx]

PONE-D-20-29379R1

Using high-definition transcranial direct current stimulation to investigate the role of the dorsolateral prefrontal cortex in explicit sequence learning

PLOS ONE

Dear Dr. Ballard,

Thank you for submitting your manuscript to PLOS ONE. After careful consideration, we feel that it has merit but does not fully meet PLOS ONE’s publication criteria as it currently stands. Therefore, we invite you to submit a revised version of the manuscript that addresses the points raised during the review process.

As you will see below, the Reviewers were mostly satisfied with the revised version of your manuscript. Reviewer #2 asks that the lack of a proper control experiment to address spatial specificity is recognized explicitly as a limitation in the discussion. Please address this comment and submit a revised version.

Please submit your revised manuscript by Mar 05 2021 11:59PM. If you will need more time than this to complete your revisions, please reply to this message or contact the journal office at plosone@plos.org. When you're ready to submit your revision, log on to <https://www.editorialmanager.com/pone/> and select the 'Submissions Needing Revision' folder to locate your manuscript file.

Please include the following items when submitting your revised manuscript:

- A rebuttal letter that responds to each point raised by the academic editor and reviewer(s). You should upload this letter as a separate file labeled 'Response to Reviewers'.
- A marked-up copy of your manuscript that highlights changes made to the original version. You should upload this as a separate file labeled 'Revised Manuscript with Track Changes'.
- An unmarked version of your revised paper without tracked changes. You should upload this as a separate file labeled 'Manuscript'.

If you would like to make changes to your financial disclosure, please include your updated statement in your cover letter. Guidelines for resubmitting your figure files are available below the reviewer comments at the end of this letter.

If applicable, we recommend that you deposit your laboratory protocols in [protocols.io](http://protocols.io/) to enhance the reproducibility of your results. [Protocols.io](http://protocols.io/)assigns your protocol its own identifier (DOI) so that it can be cited independently in the future. For instructions see: [http://journals.plos.org/plosone/s/submission-guidelines#loc-laboratory-protocols](https://urldefense.com/v3/__http://journals.plos.org/plosone/s/submission-guidelines*loc-laboratory-protocols__;Iw!!KwNVnqRv!XWsWgovHu7xxcnzVLzLfF_ejBYbom8TML1zNuuS1OWyx0emmRV3ByGZvgsKTbIRCIwM9$)

We look forward to receiving your revised manuscript.

Reviewer #1: The authors have carefully considered the questions raised by the reviewers. Overall, their responses are satisfactory, given the current circumstances.

I requested to consider adding an experiment addressing the spatial specificity of the effect observed by anodal and cathodal stimulation of the DLPC. In response, the authors “believe that involving other brain areas will obscure results”. I remain skeptical about this. After all, application of high-density stimulation is highlighted as one novel aspect of the study. This would be pointless, if the advance on spatial resolution would not also be harvested. If the authors think that adding any brain area will have deleterious effects on sequence learning, the effect seen after DLPC stimulation does not support any specific contribution to a cerebellar-frontal network. However, I understand the limitations imposed by the current pandemic. Therefore, waiving an additional experiment could be acceptable if the limitations resulting from the waiver are adequately described. I suggest to address this limitation more explicitly.

**We thank the reviewer for their thorough consideration of this point. We have noted the resulting limitation in the discussion section of the revised manuscript.**

Reviewer #2: No further comments to add. The manuscript can be accepted as it is.
